# Carbohydrate quality index and risk of non-alcoholic fatty liver disease in Iranian adults

**DOI:** 10.1186/s12902-024-01609-1

**Published:** 2024-09-20

**Authors:** Mitra Kazemi Jahromi, Niloufar Saber, Mostafa Norouzzadeh, Ghazal Daftari, Fatemeh Pourhabibi-Zarandi, Hamid Ahmadirad, Hossein Farhadnejad, Farshad Teymoori, Ammar Salehi-Sahlabadi, Parvin Mirmiran

**Affiliations:** 1 Endocrinology and Metabolism Research Center, Hormozgan University of Medical Sciences, Bandar Abbas, Iran; 2 Nutrition and Endocrine Research Center, Research Institute for Endocrine Sciences, Shahid Beheshti University of Medical Sciences, Tehran, Iran; 3 School of Medicine, Tehran University of Medical Sciences, Tehran, Iran; 4 Department of Community Nutrition, School of Nutrition and Food Sciences, Shiraz University of Medical Sciences, Shiraz, Iran; 5 Department of Nutrition, School of Public Health, Iran University of Medical Sciences, Tehran, Iran; 6 Department of Clinical Nutrition and Dietetics, Faculty of Nutrition and Food Technology, National Nutrition and Food Technology Research Institute, Shahid Beheshti University of Medical Sciences, Tehran, Iran; 7 Department of Community Nutrition, School of Nutrition and Food Science, Isfahan University of Medical Sciences, Isfahan, Iran

**Keywords:** Carbohydrate intake, Dietary fiber, Whole grain, Glycemic index, Non-alcoholic fatty liver disease

## Abstract

**Background/aim:**

In the current study, we aimed to assess the association of carbohydrate quality index (CQI) with the risk of non-alcoholic fatty liver disease (NAFLD) in Iranian adults.

**Methods:**

This case-control study was conducted on 225 newly diagnosed NAFLD patients and 450 controls, aged 20–60 years. A food frequency questionnaire was used to calculate the CQI and its components, including fiber intake, glycemic index, whole grains: total grains ratio, and solid carbohydrates: total carbohydrates ratio. Multivariable logistic regression was used to estimate the odds ratio (OR) of NAFLD across the tertile of CQI and its components.

**Results:**

The participant’s mean ± SD of body mass index and age were 26.8 ± 4.3 kg/m^2^ and 38.1 ± 8.8 years, respectively. The median (interquartile) CQI score in participants of the case and control groups was 20 (15–25) and 23 (18–28), respectively. In the multivariable-adjusted model, the risk of NAFLD decreased significantly across the tertiles of the CQI [(OR: 0.20; %95CI: 0.11–0.39), P_trend_ <0.001)]. Also, the odds of NAFLD decreased across tertiles of solid carbohydrates to total carbohydrates ratio [(OR: 0.39; 95%CI: 0.22–0.69), P_trend_ <0.001)]. However, a high dietary glycemic index (GI) was associated with increased odds of NAFLD [(OR:7.47; 95%CI: 3.89–14.33, P_trend_<0.001)]. There was no significant relationship between other CQI components, including fiber intake and whole grain/total grains and the risk of NAFLD.

**Conclusions:**

Our results revealed that a diet with a high quality of carbohydrates, characterized by higher intakes of solid carbohydrates, whole grain, and low GI carbohydrates, can be related to a reduced risk of NAFLD.

**Supplementary Information:**

The online version contains supplementary material available at 10.1186/s12902-024-01609-1.

## Introduction

Nonalcoholic fatty liver disease (NAFLD) is an important metabolic disorder that is considered an important cause of liver burden globally [1]. NAFLD spectrum includes fatty infiltration (NAFLD), nonalcoholic steatohepatitis (NASH), fibrosis, and eventually cirrhosis regardless of viral infection or excessive alcohol consumption [1, 2]. The prevalence of NAFLD is estimated at 15–30% in the general population worldwide [3]. Also, a recent investigation has reported the prevalence of NAFLD in the studied populations in Iranian society to be more than 25% [4]. Central obesity and type 2 diabetes are the main risk factors of NAFLD [5]. Moreover, a sedentary lifestyle, hypertension (HTN), and cardiovascular diseases (CVDs) are recognized as other risk factors for NAFLD progression [6–8].

Like other lifestyle factors [9], dietary factors can be considered as a modifiable detrimental or protective factor in the occurrence or development of NAFLD [10–14]. Therefore, assessing the role of nutrition in different aspects and levels, including dietary patterns, food groups, and nutrients, can help in discovering the contribution of nutrition in predicting the risk of NAFLD [10]. Carbohydrates are the main sources of diet and play a key role in human health and body metabolism [15]. While it is obvious that overconsumption of calories results in fat accumulation, additionally macro and micronutrient composition can modify the risk factors [16]. It is possible that if the portion of carbohydrates from the total daily energy intake is unusual or more than the body’s needs, it may play a role in creating an inappropriate metabolic status and accelerating liver dysfunction [16, 17]. Carbohydrate (CHO) overfeeding causes increased levels of triglyceride (TG) and very low-density lipoprotein (VLDL) and also stimulates de novo lipogenesis which can improve NAFLD [16].

Although overconsumption of carbohydrates is related to NAFLD progression, an individual component cannot thoroughly indicate the entire quality of dietary carbohydrates [18]. Hence, broader criteria that combine several single components into an integrated index represent the quality of dietary carbohydrates better [18]. Zazpe et al. suggested the carbohydrate quality index (CQI) defined by the following criteria: dietary fiber intake, whole grain to total grain ratio, glycemic index (GI), and solid carbohydrate to total carbohydrate, presuming that greater CQI provides nutrient adequacy and lowers the risk of chronic pathologies [19]. Recently, in limited studies, the possible role of CQI, as a measure to assess the quality and quantity of carbohydrates in the diet, with the risk of some metabolic diseases has been investigated, which indicating noticeable results. Some studies previously reported that a diet with a high CQI score was significantly associated with a reduced risk of CVDs, HTN [20], chronic kidney disease [21], and obesity [20]. Considering the high prevalence of NAFLD in the Iranian population and the lack of studies on the possible associations of carbohydrate quality and quantity with the risk of this disease, we aimed to investigate the relationship between CQI and its components with the risk of NAFLD in Iranian adults.

## Materials and methods

### Study population

The current study was performed in the Metabolic Liver Disease Research Center, a referral center affiliated with Isfahan University of Medical Sciences, in the framework of a case-control design. The sample size for the current study was calculated using the G power software version 3.1.9.4. Based on the previous research assessing the CQI relationship with chronic diseases including obesity, hypertension, and metabolic syndrome, we assumed at least a 20% lower risk of NAFLD for subjects in the highest tertile of CQI compared to those in the lowest one [20]. Considering the odds ratio of 0.80, type I error of 5% and study power of 80% (β = 0.20), and the ratio of controls to cases as 2, we needed a sample of 573 participants (191 cases and 382 controls). However, we recruited 225 newly diagnosed NAFLD patients and 450 controls, aged 20 to 60 years old, to keep track of any possible drop-outs. The absence of alcohol usage and other liver disease causes, along with a liver ultrasound scan that was consistent with NAFLD, served as confirmation of the diagnosis. In addition to the absence of alcohol usage and other potential liver disease etiologies, an ultrasound scan of the liver (grade II or III) ascertained an NAFLD diagnosis. The control group was selected among non-NAFLD participants based on the ultrasonography of the liver (without any signs or symptoms of hepatic steatosis). For the present study, the following criteria were considered inclusion criteria for participants: (1) not to have a special diet; (2) having no history of kidney or liver disease, diabetes, cardiovascular disease, malignancy, severe gastrointestinal disease, thyroid disorder, or autoimmune disease; and (3) not using hepatotoxic or steatogenic drugs. We excluded the individuals who completed fewer than 35 items of the food frequency questionnaire (FFQ), as well as those who reported under or over-reported daily energy intake (≤ 800 kcal/day or ≥ 4,500 kcal/day).

### Dietary assessment

The present literature evaluated the dietary intake of all participants using a validated and reproducible semi-quantitative FFQ with 168 food items (Supplementary file 1) [22]. Trained dieticians asked participants to state their mean dietary intake during the previous year by selecting one of the categories listed as follows: “never or less than once a month”, “3–4 times per month”, “once a week”, “2–4 times per week”, “5–6 times per week”, “once daily”, “2–3 times per day”, “4–5 times per day”, and “6 or more times a day”. Standard Iranian household measures were used for transforming the all-food item portion size into a gram scale [23]. The dietary energy, micronutrient, and macronutrient intakes were calculated using the United States Department of Agriculture’s (USDA) Food Composition Table (FCT) [24]. For traditional food items that are not available in the USDA FCT, we used the Iranian FCT [25]. Finally, the frequency of consumed food items was converted into a daily intake scale.

### Calculation of carbohydrate quality index

The CQI [19] was calculated by summing four components, including dietary fiber intake (g/d); glycemic index; whole grains: total grains ratio; and solid CHO (SCHO): total CHO (TCHO) ratio. In the last component, only the amount of CHO from each food was considered. For each carbohydrate-containing food, GI is described as the area under the blood glucose response curve over two hours after eating the food relative to that after consuming the equivalent amount of carbohydrate as glucose. The international table of GI and list of the GI of Iranian foods [26, 27] were used to obtain the GI value of each food item. The total dietary GI was determined as the following:

Dietary GI = [(carbohydrate content of each food item) × (number of servings/d) × (GI)] / total daily carbohydrate intake.

To compute total grains, we summed up dietary intakes of refined grains, whole grains, and their products. Liquid carbohydrates were defined by summing up fruit juice and sugar-sweetened beverage consumption. However, solid carbohydrates consist of carbohydrates from all other food sources [28]. In all components, the individuals were classified into quintiles according to the intake of the above components and then were assigned a value that ranged from 1 to 5. For the glycemic index, the participants who were in the first quintile received 5 points, and those in the fifth one were assigned 1 point. For other components, the individuals who were in the highest quintile were given 5 scores and those in the lowest one received 1 score. To compute the CQI score that varied from 4 to 20, the calculated score for four components was summed. Also, the score of each component was considered and reported separately [21].

### Assessment of other variables

Information regarding socio-demographic variables, including age, sex, smoking status, education level, family size, house acquisition, foreign travel, income, and socioeconomic status (SES), was collected via a standard demographic questionnaire [29]. Family members (≤ 4, > 4 people), education (academic and non-academic education), and house ownership or not were three variables that were considered to compute the SES score. For any of the above variables, individuals received a score of 1 if: (1) their family consisted of fewer than 4 people; (2) they had a college education; or (3) they owned a home. The final SES score was calculated by summing up the scores of these variables. Individuals who had 0 or 1, 2, and 3 were classified as low, moderate, and high SES, respectively. The International Physical Activity Questionnaire (IPAQ) was used in face-to-face interviews to evaluate subjects’ levels of physical activity [30], and all results were represented as metabolic equivalents per week (METs/week) [31, 32]. The body weight of participants was measured using digital scales (model 707, Seca, Hamburg, Germany) with an accuracy of up to 100 g with light clothes and without shoes. We used a stadiometer (model 208 Portable Body Meter Measuring Device; Seca) with a minimum of 0.5 cm in a standing position without shoes to measure individuals’ height. Body mass index (BMI) was computed as weight (kg) divided by height (m^2^).

### Statistical analysis

Data analysis was conducted using SPSS, version 21 (Statistical Package for Social Sciences). Kolmogorov–Smirnov test and histogram chart were used to evaluate the data normality. Baseline characteristics and dietary intakes of the study population were reported as mean ± SD or median (25–75 interquartile range) and frequency (percentages) for quantitative and qualitative variables, respectively. Differences in variables between the case and control groups were compared using the chi-square and independent sample t-test for categorical and continuous variables, respectively. Participants were divided into tertiles according to CQI and its components. The P-trend of continuous and categorical variables across tertiles of CQI and its components were evaluated using linear regression and the chi-square test. Multivariable logistic regression was used to evaluate the odds ratio (OR) with a 95% confidence interval (CI) of NAFLD in each tertile of CQI and its components according to three models including model 1 (the crude model), model 2 (adjusted for age and sex), and model 3 (adjusted for model 1 and BMI, smoking, physical activity, SES, and energy intake). A *P*-value < 0.05 was used as the statistical evaluation tool.

## Results

The participant’s (53% male) mean ± SD of BMI and age were 26.8 ± 4.3 kg/m^2^ and 38.1 ± 8.8 years, respectively. The median (interquartile range) of CQI score in participants of the case and control groups was 20 (15–25) and 23 (18–28), respectively. Participants’ characteristics across the tertiles of CQI are presented in Table 1. Our findings showed that the mean age was increased across tertiles of CQI (*P* < 0.05). However, there is no significant difference in other demographic data, socio-economic status, BMI, smoking, and physical activity across tertiles of CQI. Dietary intakes of energy, protein, fiber, whole-grain/total-grain, SCHO /TCHO, and CQI score were increased in participants across tertiles of CQI (*P* < 0.05), whereas the intake of fats was decreased across tertiles of CQI (*P* < 0.05).

**Table 1:** Population characteristics based on the tertiles of carbohydrate quality index

	T_1_ (*n* = 247)	T_2_ (*n* = 230)	T_3_ (*n* = 198)	*P*-trend
**Demographic data**				
Age, (year)	36.26 (8.01)	38.97 (9.27)	39.48 (8.99)	< 0.001
Sex (male)	55.1	50.4	53.5	0.591
Body mass index (Kg/m^2^)	26.83 (3.98)	27.37 (4.80)	26.26 (4.06)	0.191
Physical activity (MET/h/week)	1484.7 (853.7)	1438.6 (954.7)	1362.6 (823.0)	0.147
Smoking (yes)	5.7	3.5	3.0	0.324
Education level (Bachelor and higher)	46.6	47.8	46.0	0.924
Socio economic status				0.218
Low	28.3	23.0	33.8	
Middle	36	43.5	34.8	
High	35.6	33.5	31.3	
**Dietary intake**				
Energy intake (Kcal/d)	2018.2 (581.1)	2221.3 (607.1)	2466.2 (603.0)	< 0.001
Carbohydrate (% Kcal)	55.50 (7.54)	56.13 (6.39)	56.19 (6.45)	0.285
Protein (% Kcal)	12.82 (2.20)	13.35 (2.34)	13.66 (2.26)	< 0.001
Fat (% Kcal)	31.66 (7.74)	30.50 (6.22)	30.13 (6.27)	0.018
Fiber (g / 1000Kcal)	13.58 (6.78)	17.25 (7.40)	18.12 (6.34)	< 0.001
Glycemic index	66.14 (7.45)	60.39 (6.69)	53.77 (6.61)	< 0.001
Whole-grain/total-grain	0.08 (0.06)	0.19 (0.14)	0.36 (0.18)	< 0.001
SCHO /TCHO	0.97 (0.02)	0.98 (0.02)	0.99 (0.009)	< 0.001
Carbohydrate quality index	14.80 (3.15)	22.35 (1.70)	30.54 (3.55)	< 0.001

Data were expressed as mean (SD) and percent (%) for continuous and categorical variables, respectively

Table 2 indicates the study population characteristics based on the case and control groups. Compared to the controls, NAFLD patients were more likely to be low active, highly smoked, and have higher BMI. Also, % of high SES in subjects in the case group was higher than in the control group. However, there are no significant differences between age, sex, and educational level of NAFLD patients and controls (*P*-value > 0.05). Based on Table 2, participants in the control group had a higher score of CQI and SCHO /TCHO compared to NAFLD patients (*P*-value < 0.05). However, NAFLD patients had a higher dietary energy intake, fiber intake, and dietary GI rather than controls (*P*-value < 0.05). There was no significant difference in mean carbohydrate (% kcal), fat (% kcal), protein (% kcal), and whole-grain/total-grain between the case and control groups.

**Table 2:** Study population characteristics based on the case and control groups

Variables	Non-NAFLD (*n* = 450)	NAFLD (*n* = 225)	*P*-value*
**Demographic data**			
Age, (year)	37.88 ± 8.91	38.63 ± 8.71	0.296
Sex (male)	51.8	55.6	0.354
Body mass index (Kg/m^2^)	24.99 ± 3.09	30.56 ± 4.02	**< 0.001**
Physical activity (MET/min/week)	1590.3 ± 949.4	1119.0 ± 616.3	**< 0.001**
Smoking (yes)	2.7	7.1	**0.006**
Socio economic status			**< 0.001**
Low	31.6	21.3	
Middle	40.4	33.8	
High	28.0	44.9	
**Dietary intake**			
Energy intake (Kcal/d)	2170.5 ± 625.4	2315.4 ± 606.5	**0.004**
Carbohydrate (% Kcal)	55.90 ± 6.56	55.96 ± 7.41	0.913
Fat (% Kcal)	30.83 ± 6.54	13.25 ± 2.21	0.939
Protein (% Kcal)	13.25 ± 2.21	13.24 ± 2.46	0.924
fiber (g / 1000Kcal)	15.86 ± 6.46	16.77 ± 8.33	**0.013**
Glycemic index	58.74 ± 8.11	64.18 ± 8.29	**< 0.001**
Whole-grain/total-grain	0.21 ± 0.18	0.19 ± 0.15	0.055
SCHO /TCHO	0.98 ± 0.01	0.97 ± 0.02	**< 0.001**
Carbohydrate quality index	22.93 ± 7.04	20.12 ± 6.46	**< 0.001**

Data were expressed as mean ± SD and percent (%) for continuous and categorical variables, respectively

* *P*- Values were determined using the independent-samples t-test and the chi-square test for continuous and categorical variables

The ORs (95%CI) of NAFLD across tertiles of CQI and its components are presented in Table 3. In the age and sex-adjusted model, the odds of NAFLD were decreased across tertiles of CQI (OR: 0.35; 95%CI: 0.23–0.54, P-trend < 0.001). Also, in the multivariable model, after adjusting for age, sex, BMI, smoking status, physical activity, SES, and dietary energy intake, a higher score of CQI was associated with reduced odds of NAFLD (OR: 0.20; 95%CI:0.11-0.39,P-trend <0.001) .

**Table 3:** Odds ratio (95% CI) of NAFLD across tertiles of CQI and its components

Dietary indices	OR (95% CI) of NAFLD			*P* trend*
	Tertile 1	Tertile 2	Tertile 3	
**Carbohydrate quality index**				
Median score	15	22	30	
Case/Total	103/247	80/230	42/198	
Crude model	1.00 (Ref)	0.74 (0.51, 1.08)	0.37 (0.24, 0.57)	< 0.001
Model 1*	1.00 (Ref)	0.71 (0.49, 1.04)	0.35 (0.23, 0.54)	< 0.001
Model 2^†^	1.00 (Ref)	0.44 (0.25, 0.76)	0.20 (0.11, 0.39)	< 0.001
**Glycemic index**				
Median score	52.43	60.51	67.93	
Case/Total	41/225	71/224	113/226	
Crude model	1.00 (Ref)	2.08 (1.34, 3.23)	4.48 (2.92, 6.88)	< 0.001
Model 1*	1.00 (Ref)	2.08 (1.34, 3.23)	4.53 (2.95, 6.96)	< 0.001
Model 2^†^	1.00 (Ref)	2.77 (1.46, 5.25)	7.47 (3.89, 14.33)	< 0.001
**Fiber intake**				
Median score	20.55	32.01	51.83	
Case/Total	65/225	75/225	85/225	
Crude model	1.00 (Ref)	1.23 (0.82, 1.83)	1.49 (1.00, 2.21)	0.048
Model 1*	1.00 (Ref)	1.22 (0.82, 1.83)	1.46 (0.98, 2.17)	0.063
Model 2^†^	1.00 (Ref)	1.04 (0.59, 1.85)	1.06 (0.56, 1.99)	0.865
**Whole grain/total grains**				
Median score	0.05	0.15	0.37	
Case/Total	77/225	77/224	71/226	
Crude model	1.00 (Ref)	1.00 (0.68, 1.47)	0.88 (0.59, 1.30)	0.482
Model 1*	1.00 (Ref)	1.00 (0.67, 1.47)	0.86 (0.57, 1.27)	0.413
Model 2^†^	1.00 (Ref)	1.26 (0.72, 2.20)	0.58 (0.33, 1.03)	0.027
**SCHO /TCHO**				
Median score	0.97	0.98	0.99	
Case/Total	107/225	61/224	57/226	
Crude model	1.00 (Ref)	0.41 (0.27, 0.61)	0.37 (0.25, 0.55)	< 0.001
Model 1*	1.00 (Ref)	0.40 (0.27, 0.60)	0.35 (0.23, 0.53)	< 0.001
Model 2^†^	1.00 (Ref)	0.54 (0.30, 0.94)	0.39 (0.22, 0.69)	0.001

Abbreviations: NAFLD, Nonalcoholic fatty liver disease; CQI, Carbohydrate quality index; SCHO/ TCHO, Solid carbohydrates/Total carbohydrates ratio

*Model 1: adjusted for age and sex

^†^ Model 2: adjusted for model 1 and body mass index, smoking, physical activity, socio-economic status, and dietary intake of energy

Also, the higher dietary GI, as a negative component of CQI, was related to increased odds of NAFLD based on the age and sex-adjusted model (OR: 4.53; 95%CI: 2.95, 6.96) and multivariable model (OR: 7.47; 95%CI: 3.89, 14.33, P-trend < 0.001). Our finding also suggested that in the age and sex-adjusted model, the odds of NAFLD were decreased across tertiles of SCHO /TCHO (OR: 0.35; 95%CI: 0.23–0.53, P-trend < 0.001). In the multivariable-adjusted model, the negative relationship between SCHO / TCHO (OR:0.39; %95 CI:0.22–0.69, P-trend < 0.001) and the risk of NAFLD has remained significant. Furthermore, no significant association was observed between higher fiber intake and whole grain/total grains and NAFLD odds.

## Discussion

Our case-control study among Iranian adults revealed that a higher quality of carbohydrates, as measured by CQI, was significantly associated with decreased risk of NAFLD, after adjusting for potential confounding factors, including age, sex, body mass index, smoking, physical activity, socio-economic status, and dietary intake of energy. A higher ratio of whole grain/total grains and SCHO/TCHO was also associated with a decreased risk of NAFLD. However, we found that consuming higher GI carbohydrates was significantly associated with an increased risk of NAFLD.

To our knowledge, this is the first study to investigate the association between CQI and the risk of NAFLD. Our findings provide important insights into the role of carbohydrate quality in the development of NAFLD and contribute to the growing body of evidence on the health benefits of high-quality carbohydrate intake. The significant association between CQI and NAFLD risk, as well as the observed associations between whole grain/total grains ratio and SCHO/TCHO ratio and NAFLD risk, highlight the importance of not only the quantity but also the quality of carbohydrate intake in the prevention of NAFLD. Our study also adds to the existing knowledge by highlighting the detrimental effects of high GI carbohydrates on NAFLD risk. Overall, our study underscores the need for further research to explore the complex relationship between carbohydrate quality and NAFLD risk, particularly in diverse populations where dietary patterns and disease prevalence may differ.

Although no study has yet investigated the association between CQI and the risk of NAFLD, our results are comparable with findings from prior research that have explored the possible associations between the quality and quantity of carbohydrate intake and the risk of chronic diseases. Zazpe et al. found that higher scores of CQI were associated with a lower risk of CVDs incidence [33]. Kim et al. reported that a higher CQI was inversely associated with the risk of obesity and HTN [34]. Another study conducted on individuals with type 2 diabetes showed that a higher CQI was associated with lower odds of metabolic syndrome [35]. Furthermore, Teymoori et al. observed an inverse relationship between CQI and the risk of chronic kidney disease [21].

While no study has specifically investigated the relationship between CQI and NAFLD risk, previous research has explored the association between carbohydrate intake and the risk of NAFLD. For example, some studies have reported that high intake of refined carbohydrates and simple sugars, which are often high in GI, are associated with an increased risk of NAFLD [36], while diets that are rich in high-quality carbohydrates, such as whole grains and low GI carbohydrates, are associated with a lower risk of NAFLD [37, 38], which are consistent with our results. Our study further supports these findings by showing that a higher CQI, which reflects a higher intake of whole grains, lower GI, and solid carbohydrates is associated with a lower risk of NAFLD.

Although previous research has suggested that fiber intake may have a protective effect against NAFLD [39], our study did not find a significant association between fiber intake and NAFLD risk. One possible explanation for this is that the effect of fiber on NAFLD risk may be indirect, and mediated by other dietary and lifestyle factors. Additionally, the role of fiber in NAFLD may also depend on the type and source of fiber [40], as well as the individual’s gut microbiota [41], which can affect the fermentation and absorption of fiber in the gut. Also, differences in general individual characteristics, such as gender, age, race, and dietary patterns as well as differences in study design and adjustment of confounding factors, may contribute to the controversies in the findings of previous investigations. Therefore, further research is needed to better understand the complex relationship between fiber intake, dietary patterns, gut microbiota, and NAFLD risk.

Diets that are rich in high-quality carbohydrates, as assessed by CQI, are typically characterized by a higher consumption of whole grains, fiber, a lower GI, and a lower intake of simple carbohydrates. These dietary features have the potential to lower the risk of NAFLD through various mechanisms. For example, previous studies have reported that a higher intake of whole grains is associated with a lower risk of liver cancer and liver disease mortality [40], potentially due to their rich source of dietary fiber, resistant starch, and oligosaccharides [42, 43] that have been linked to a range of beneficial effects on liver health, including reducing blood glucose and insulin sensitivity, lowering liver fat content, and regulating inflammation [44–46]. These benefits could be mediated by changes in gut microbiota and the gut-liver axis, which play a critical role in maintaining liver health [47]. Moreover, animal and human studies have suggested that high GI diets can promote fat accumulation in liver cells and contribute to the development of hepatic steatosis [37, 48]. This may be because high GI foods can cause rapid spikes in blood sugar levels, which can trigger the “de novo” synthesis of fatty acids and stimulate the accumulation of fat in liver cells [48]. Similarly, the consumption of liquid and simple carbohydrates, such as sugar-sweetened beverages, can lead to a rapid increase in insulin and glucose levels in the bloodstream, which may contribute to the development of insulin resistance and further increase the risk of NAFLD [49, 50].

Our study is the first to investigate the association between the CQI and the risk of NAFLD in Iranian adults, filling an important gap in the literature. Also, the use of a validated and reproducible FFQ and a validated physical activity questionnaire enhances the accuracy and reliability of our results. However, it is important to acknowledge the limitations of our study. Firstly, the case-control design of the study does limit our ability to establish a clear causal relationship. Secondly, using a questionnaire to collect data on dietary intake and physical activity is subject to measurement errors, but we attempted to minimize these errors by using a validated questionnaire specifically designed for our study population. Thirdly, the present hospital-based study was conducted in a particular metabolic liver disease center in Iran, which may limit its generalizability. Finally, in the current study, NAFLD diagnosis was based on ultrasonography, however, in diagnosing NAFLD in individuals, biopsy of the liver and magnetic resonance imaging (MRI) are considered gold standard tests due to their high accuracy and ability to provide detailed information about liver tissue structure. It should be noted that biopsy and MRI tests have limitations and complications, such as being expensive and invasive in the case of biopsy, or being less accessible due to high costs in some areas for MRI test; however, ultrasonography test is a noninvasive and easily accessible test, and is the applicable and reliable imaging technique of choice for screening for fatty liver in clinical and population settings [51, 52].

## Conclusions

Our results provide evidence that a diet with a high score of CQI, determined by higher consumption of complex carbohydrates and lower consumption of liquid carbohydrates and high GI foods, is associated with a decreased risk of NAFLD in Iranian adults. These findings highlight the importance of the quality and quantity of carbohydrate intake in the prevention of NAFLD and suggest that interventions aimed at improving carbohydrate quality may be beneficial in reducing the burden of NAFLD. However, future epidemiological studies, particularly prospective studies with larger sample sizes and longer follow-up periods, are recommended to confirm the relationship between CQI and NAFLD risk.

## Electronic supplementary material

Below is the link to the electronic supplementary material.

Supplementary Material 1

## Data Availability

The datasets analyzed in the current study are available from the corresponding author on reasonable request.

## Abbreviations

BMI: Body mass index
CQI: Carbohydrate quality index
CVDs: Cardiovascular diseases
FCT: Food Composition Table
FFQ: Food frequency questionnaire
GI: Glycemic index
HTN: Hypertension
IPAQ: International Physical Activity Questionnaire
MET: Metabolic equivalents per week
NAFLD: Non-alcoholic fatty liver disease
NASH: Non-alcoholic steatohepatitis
TG: Triglyceride
SES: Socioeconomic status
USDA: United States Department of Agriculture
VLDL: Very low-density lipoprotein
