# Supplementary material for: Carbohydrate quality index and risk of non-alcoholic fatty liver disease in Iranian adults
Source: BMC Endocr Disord. 2024 Sep 20;24:195. doi: 10.1186/s12902-024-01609-1 (PMC11414208; doi:10.1186/s12902-024-01609-1)
Supplement: Supplementary file 1 — Supplementary Material 1 [file 12902_2024_1609_MOESM1_ESM.pdf]

Supplementary file 1

In the Name of God

Research Institute for Endocrine Sciences

Shahid Beheshti University of Medical Sciences,

Food Frequency Questionnaire (FFQ) "Tehran Lipid and Glucose Study"

Name: Code: Age:

NO.	Food items	Portion size	How often				Comments
			Daily	Weekly	Monthly	Yearly	
1	Lavash bread	1 slice					
2	Barbari bread	1 slice					
3	Sangak bread	1 slice					
4	Taftoon bread	1 slice					
5	Baguette bread	1 small					
6	Toasted bread	1 slice					
7	Cooked rice	1 plate					
8	Cooked pasta	1 plate					
9	Potato	1 medium					
10	French fries	10 number					
11	Baked vermicelli (soup noodle)	1 cup					
12	Ash noodle (Reshteh)	1 cup					
13	wheat flour	1 cup					
14	Cookies	1 number					
15	Crackers	1 number					
16	Yazdi cake	1 number					
17	Homemade cake	1 medium slice					
18	Other cakes	1 number					
19	Corn	1 medium					
20	Barley	1 tsp					
21	Bulgur	1 cup					
22	Lentil	1 cup					
23	Beans	1 cup					
24	Chickpea	1 cup					
25	Broad bean	1 cup					
26	Soy bean	1 cup					
27	Mung bean	1 cup					
28	Split chickpea	1 cup					
29	Beef	1 slice					
30	Lamb meat	1 slice					

31	Ground beef	1 tablespoon					
32	Chicken with skin	1 medium piece					
33	Chicken without skin	1 medium piece					
34	Fish (except tuna)	1 medium piece (one palm full)					
35	Tuna (canned)	1/2 cans					
36	Hamburger	1 number					
37	Sausage	1 number (Germany * cocktails *)					
38	Beef ham	1 slice					
39	Heart, liver and kidney	1 slice					
40	Egg	1 number					
41	Tripe and Rennet	1 piece					
42	Tongue	1 whole number					
43	Brain	1 whole number					
44	Kalle-Kind of organ meat	1 number					
45	Pache-kind of organ meat	1 number					
46	Pizza	1 slice					
47	Skimmed milk	1 cup					
48	Low-fat milk (< 2%)	1 cup					
49	Whole milk (> 2%)	1 cup					
50	Cacao milk	1 cup					
51	Chocolate milk	1 cup					
52	Concentrated yogurt	1 tablespoon					
53	Plain yogurt	1 cup					
54	Full fat yogurt	1 cup					
55	Cream yogurt	1 tablespoon					
56	Cheese	1 slice					
57	Cream cheese	1 slice					
58	Dough	1 cup					
59	Cream	1 tablespoon					
60	Traditional ice cream	½ cup					
61	Non-traditional ice cream	½ cup					
62	Butter	1 slice					
63	Margarine	1 slice					
64	Kashk	1 tablespoon					
65	Shredded lettuce	1 cup					

66	Tomato	1 medium					
67	Cucumber	1 medium					
68	Fresh Herbs	1 small plate					
69	Cooked greens	1 cup					
70	Pumpkin	1 medium					
71	Squash	1 medium					
72	Eggplant	1 medium					
73	Celery	1 cup					
74	Green peas	1 cup					
75	Green beans	1 cup					
76	Raw carrots	1 medium					
77	Cooked carrots	1 medium					
78	Garlic	1 clove					
79	Raw onion	1 small					
80	Fried onions	1 tablespoon					
81	Cabbage varieties	1 cup					
82	Bell peppers	1 medium					
83	Raw spinach	20 medium leaves					
84	Cooked spinach	1 cup					
85	Turnip	1 medium					
86	Small green pepper	1 medium					
87	ketchup	1 tablespoon					
88	Pickles in vinegar	1 tablespoon					
89	Salted vegetables	1 tablespoon					
90	Pickled cucumber	1 medium					
91	Cantaloupe	¼ number					
92	Melon	1 medium slice					
93	Watermelon	1 medium slice					
94	Pear	1 medium					
95	Apricot	1 medium					
96	Cherries	10 number					
97	Apple	1 medium					
98	Peach	1 medium					
99	Nectarine	1 medium					
100	Green plum	1 medium					
101	Fresh figs	1 medium					
102	Dried figs	1 medium					
103	Grape	1 medium bunch					
104	Kiwi	1 medium					
105	Grapefruit	1 medium					
106	Orange	1 medium					
107	Persimmon	1 medium					
108	Tangerine	1 medium					
109	Pomegranate	1 medium					

110	Date	1 medium					
111	Plums (yellow and red)	1 medium					
112	Sour cherry	10 number					
113	Strawberry	3 number					
114	Banana	1 medium					
115	Sweet lemon	1 medium					
116	Lemon	1 medium					
117	Grapefruit juice	1 cup					
118	Orange juice	1 cup					
119	Apple juice	1 cup					
120	Cantaloupe juice	1 cup					
121	Cranberry	1 cup					
122	Fresh Pineapple	1 cup					
123	Canned Pineapple	1 cup					
124	Raisins	1 tablespoon					
125	Persian cantaloupe	1 cup					
126	Fresh mulberry	10 number					
127	Dried mulberry	20 number					
128	Dried Peach	10 number					
129	Dried apricot	10 number					
130	Green Olive	10 number					
131	Canned fruits	1 can					
132	Hydrogenated oils	1 tablespoon					
133	Liquid Oil	1 tablespoon					
134	Olive oil	1 tablespoon					
135	Tallow (fat)	1 medium slice					
136	Ghee	1 tablespoon					
137	Mayonnaise	1 tablespoon					
138	Peanut	20 number					
139	Almond	10 number					
140	Walnut	1 number					
141	Pistachios	10 number					
142	Hazelnut	10 number					
143	Seeds (watermelon, pumpkin, sunflower)	1 cup					
144	Sugar cube	10 number					
145	Sugar	1 teaspoonful					
146	Honey	1 teaspoonful					
147	Jams (by type)	1 tablespoon					
148	Soft drinks	1 cup					
149	Pastries	1 medium					
150	Creamy pastries	1 medium					
151	GAZ	1 medium					
152	Candy	1 number					

153	SOHAN	1 Piece					
154	Puff	1 pack					
155	Chocolate	1 number					
156	Caramel cream	1 tablespoon					
157	Tea	1 cup					
158	Salt	1 tablespoon					
159	Broth(only water)	1 cup					
160	Chips	1 pack					
161	Coffee	1 cup					
162	Lemon juice	1 teaspoonful					
163	NABAT	1 medium					
164	Mushroom	½ cup					
165	Homemade HALVA	1 tablespoon					
166	Halva Ardeh	¼ number					
167	NOGHL	10 number					
168	Donuts	1 number					
